# Role of Oxygen and Halogen Functionalization in Tuning the Surface Properties of Zr_3_C_2_T_2_ MXene for Lithium Storage: A Density Functional Theory Study

**DOI:** 10.3390/ma18061237

**Published:** 2025-03-11

**Authors:** Hui Li, Zhengyang Xie, Tianwei Gao, Jinyi Liu, Wenke Lu, Yue Liu, Shouwei Wang

**Affiliations:** School of Materials Science and Engineering, Chang’an University, Xi’an 710064, China; 2022131042@chd.edu.cn (Z.X.); 2022231101@chd.edu.cn (T.G.); 2023131040@chd.edu.cn (J.L.); luwenke420@gmail.com (W.L.); liuyue393628@outlook.com (Y.L.); wangshouwei@chd.edu.cn (S.W.)

**Keywords:** Zr_3_C_2_ MXene, two-dimensional materials, DFT, lithium storage

## Abstract

We constructed computational models of bare Zr_3_C_2_ and surface-functionalized Zr_3_C_2_T_2_ (T = O, S, F, Cl), and utilized first-principles calculations to systematically explore the effects of these surface-functionalized groups on the structural stability, electronic properties, and lithium storage performance of Zr_3_C_2_T_2_. Compared to halogen functional groups (e.g., F, Cl), the structure and electronic properties of Zr_3_C_2_ are more profoundly influenced by oxygen group functional elements (O, S). The formation energy of Zr_3_C_2_T_2_ (T = O, S) functionalized by the same periodic oxygen group elements is lower than that of Zr_3_C_2_T_2_ (T = F, Cl) functionalized by the same periodic halogens. Regarding electronic properties, the oxygen and sulfur functional groups have strong hybridization with Zr_3_C_2_ in the valence band and generate a new band structure, which makes the DOS move toward the conduction band. The adsorption energy calculations reveal that lithium ions exhibit stable adsorption on bare Zr_3_C_2_ and O/S-functionalized Zr_3_C_2_T_2_ surfaces, whereas no stable adsorption occurs on Zr_3_C_2_F_2_ or Zr_3_C_2_Cl_2_. In terms of adsorbing lithium atoms, bare Zr_3_C_2_ tends to adsorb at the HCP position, while Zr_3_C_2_O_2_ and Zr_3_C_2_S_2_ tend to adsorb at the CCP position. First-principles calculations indicate distinct theoretical lithium storage capacities for Zr_3_C_2_-based materials: monolayer adsorption yields capacities of 180.13 mAh/g (bare Zr_3_C_2_), 162.64 mAh/g (Zr_3_C_2_O_2_), and 148.20 mAh/g (Zr_3_C_2_S_2_); bilayer adsorption significantly increases these values to 360.25, 325.29, and 296.41 mAh/g, respectively.

## 1. Introduction

With the rapid development of new energy vehicles and intelligent electronic devices, it is crucial to develop more efficient energy storage devices [1]. Among many energy storage solutions, lithium-ion batteries (LIBs) are the optimal choice due to their high energy density, rapid charging/discharging capabilities, and long cycle life [2]. The ability of LIBs to store and release energy is primarily governed by the reversible cycling of lithium ions within electrode materials during charge/discharge cycles [3]. Conventional graphite anodes are constrained by their limited theoretical capacity and poor rate performance, which fall short of the stringent requirements for next-generation LIBs [4]. Consequently, the development of advanced anode materials has become a central focus in LIB research [5]. Among the numerous candidate electrode materials, two-dimensional (2D) materials have received extensive attention due to their unique layered structures [6]. The currently reported 2D materials, including graphene [7,8,9,10], transition metal disulfide [11,12,13], phosphorene [14,15,16,17], and MXene [18,19,20,21,22,23,24], are promising electrode materials for lithium-ion batteries. Although graphene, transition metal disulfide, and phosphorene can be used as electrode materials, their low electrical conductivity and lithium-ion transport rate greatly affect the performance of the batteries [25,26,27]. Since the successful preparation of MXene in 2011 [28], it has gradually attracted significant research attention because of low energy barriers and large interlayer spaces for metal ion diffusion [29,30].

MXene is typically synthesized by the etching of “A” layers from layered ternary MAX phases using hydrofluoric acid or a solution composed of a mixture of hydrochloric acid and lithium fluoride. The chemical formula of MXene is usually expressed as M_n+1_X_n_T_x_ (n = 1~3) [31], where M represents a metallic element, mainly transition metals such as Ti, Zr, and Hf; X stands for either C or N; and T denotes surface functional groups, such as -OH and halogen groups (-F, -Cl) [32]. This material is composed of metal carbides or nitrides and has a two-dimensional layered structure. The applications of MXene in the field of energy storage have received increasing attention since Naguib et al. [28] reported that MXene materials can be used as electrode materials for lithium-ion batteries. Currently reported MXenes for lithium-ion battery electrodes include Ti_3_C_2_ [33,34,35], Mo_2_C [36,37], Nb_2_C [38,39], V_2_C [39,40,41], Mo_2_CS_2_ [42], the double-transition metal MXene Mo_2_TiC_2_ [43], MoWC [44], etc. Extensive reports have shown that the properties of MXenes strongly depend on the surface functional groups [45,46]. Li et al. [47] analyzed the effect of different groups (O, H, and OH) on the lithium storage properties of Ti_3_C_2_ using the DFT method. The results revealed that O reduced the adsorption energy of lithium atoms, while F limited the adsorption of Li atoms, and the Li atoms could not even stably adsorb on the Ti_3_C_2_H_2_ and Ti_3_C_2_(OH)_2_ surfaces. Li et al. [48] modified the lithium storage performance of Ti_2_C by introducing eight different doping atoms. The results showed that different doping atoms altered the d-band center of the material, thereby affecting the adsorption energy of lithium on the MXene surface. Using V as the doping atom resulted in the best adsorption energy, open circuit voltage, and minimum diffusion energy. Abdelsalam et al. [49] used DFT to calculate the adsorption of lithium on Zr_2_C and Zr_2_CO_2_ MXenes. The results demonstrated that lithium can be stably adsorbed on Zr_2_C and Zr_2_CO_2_, with a theoretical capacity higher than graphite, making it a favorable candidate for lithium-ion battery electrodes.

A new Zr_3_C_2_T_x_ MXene was successfully synthesized [50] by selectively etching Al_3_C_3_ from Zr_3_Al_3_C_5_ and was then theoretically [51] investigated. Recently, computational insights into the influence of surface functionalization groups on Zr_3_C_2_T_2_ (T = O, F) [52] for sodium-sulfur batteries and Zr_3_C_2_T_2_ (T = H, Si, P, S, Se) [53] for lithium-ion batteries were studied. Khan [54] reviewed recent advances in the synthesis, properties, and novel applications of Zr_3_C_2_T_2_. Although substantial progress has been made in understanding functional group effects on MXenes, the influence of F and Cl surface functional groups on the structure, electronic behavior, and other properties of Zr_3_C_2_ MXenes still needs further exploration.

In this paper, the effects of surface terminals belonging to the oxygen (T = O, S) and halogen (T = F, Cl) groups on Zr_3_C_2_ were comparatively studied. We first constructed and optimized Zr_3_C_2_ and functionalized Zr_3_C_2_T_2_ (T = O, S, F, Cl), and then calculated the formation energies of the surface functional terminals at potential sites. At last, the effect of the functional groups (O, S, F, Cl) on the structural, electronic, and lithium storage properties of Zr_3_C_2_ MXene was studied.

## 2. Computing Method

The DFT calculations were performed using the CASTEP [55] module in Materials Studio software (Version 7.0). The Ultrasoft pseudopotentials method [56] and the generalized gradient approximation (GGA) with the Perdew–Burke–Ernzerhof (PBE) function [57] were employed during the calculations. Atomic pseudopotentials of Zr, C, O, S, F, and Cl were formed separately using electronic structures of 4d^2^5s^2^, 2s^2^2p^2^, 2s^2^2p^4^, 3s^2^3p^4^, 2s^2^2p^5^, and 3s^2^3p^5^. An 11 × 11 × 1 k-point grid for the Brillouin zone integration was generated by using the Monkhorst–Pack sampling method [58]. The Broyden–Fletcher–Goldfarb–Shanno (BFGS) method [59] was used for geometry optimization. The following parameters were set: an energy cutoff value of 450 eV, an energy convergence tolerance of 5.0 × 10^−6^ eV, a maximum force convergence tolerance of 0.01 eV/Å, a maximum stress convergence tolerance of 0.02 GPa, and a maximum displacement deviation less than 5.0 × 10^−4^ Å.

## 3. Results and Discussion

### 3.1. Structural Properties

**Figure 1:**
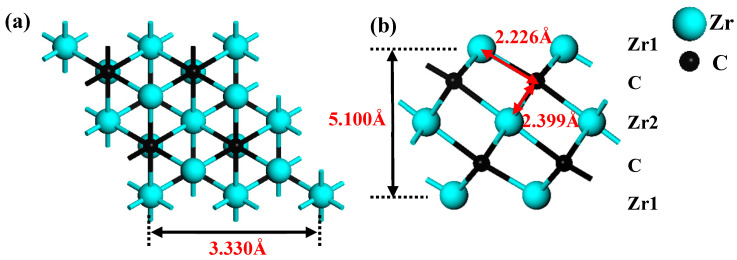
Crystal structures of 2 × 2 × 1 supercell of Zr_3_C_2_: (**a**) top view and (**b**) side view.

In this paper, the initial MAX material Zr_3_AlC_2_ came from the Material Project database [60]. The hexagonal Zr_3_C_2_ structure is obtained by “etching” Al atom layers from Zr_3_AlC_2_. The crystal structures of Zr_3_C_2_ are shown in Figure 1a,b. The optimized lattice parameter of bare Zr_3_C_2_ is 3.330 Å (close to 3.349 Å [61]), and the thickness of the monolayer is 5.100 Å, which is labeled in Figure 1a,b. The functional groups have three potential adsorption sites on the surface of the MXene: a top (TOP) site over the first layer of Zr1 atoms; a hexagonal close-packed (HCP) site over C atoms; and a cubic close-packed (CCP) site over the second layer of Zr2 atoms. The TOP site is usually a coordination atom that enhances reactivity and plays a crucial role in adsorption processes that require bond activation, while HCP and CCP are determined by the arrangement of sub-surface atoms and differ in coordination and symmetry: HCP is aligned with deep atoms, while CCP is not aligned with deep atoms. These changes affect the binding strength and selectivity, guiding the adsorption reaction pathway. By adjusting the distribution of these sites through crystal surface engineering or nanostructures, the application of materials in energy storage, stability, and catalysis can be optimized. According to the work of Li et al. [62], the Zr_3_C_2_T_2_ (T = O, S, F, Cl) models containing functional groups were constructed.

**Figure 2:**
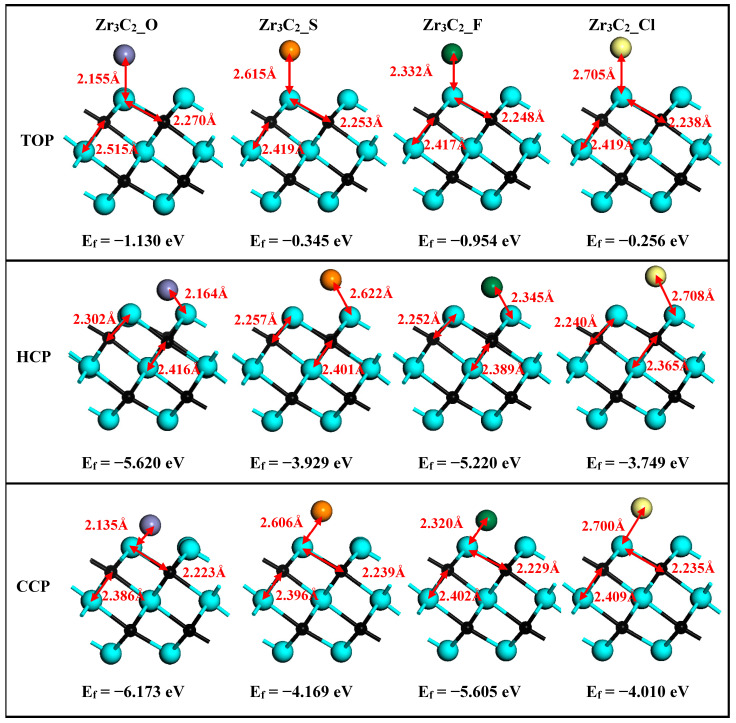
The optimized structures of Zr_3_C_2_T_2_ (T = O, S, F, Cl) with the HCP, CCP, and TOP sites.

After structural relaxation, the structures, bond lengths of d_Zr1–T_ (between the surface Zr1 and functionalized terminal atoms), d_Zr1–C_ (between the surface Zr1 and C atoms), and d_Zr2–C_ (between the middle Zr2 and C atoms) of Zr_3_C_2_T_2_ (T = O, S, F, Cl) are shown in Figure 2. It is shown in Figure 2 that the lattice constants of Zr_3_C_2_T_2_ (T = O, S, F, Cl) have been slightly changed compared to bare Zr_3_C_2_ in Figure 1. Compared with d_Zr1–C_ = 2.226 Å and d_Zr2–C_ = 2.399 Å in the bare Zr_3_C_2_, the bond length d_Zr2–C_ (2.365–2.419 Å) remains nearly constant, while the d_Zr1–C_ (2.223–2.302 Å) increases significantly in Zr_3_C_2_T_2_ (T = O, S, F, Cl). Furthermore, comparative analysis reveals distinct chemical trends: the bond lengths between the S or Cl and Zr atoms are significantly longer than those of the O or F and Zr atoms, and the bond length between the halogen functional groups (F, Cl) and Zr_3_C_2_ is longer than the counterpart between the oxygen group elements (O, S) and Zr_3_C_2_. The atomic size and atomic electronegativity may cause these discrepancies. Compared with the HCP and TOP positions, the CCP position has the shortest bond length with Zr_3_C_2_, making it the most stable site for the functional group.

To analyze the thermodynamic stability of functional groups at the HCP, CCP, and TOP positions on the Zr_3_C_2_ surface, the formation energies are calculated using Equation (1) [47]:

(1) $${\Delta E}_{f}=E_{tot}\left({Zr}_{3}C_{2}T_{2}\right)-E\left({Zr}_{3}C_{2}\right)-E\left(T_{2}\right)$$

where *E*_tot_(Zr_3_C_2_T_2_) refers to the total energy associated with Zr_3_C_2_T_2_; *E*(Zr_3_C_2_) and *E*(T_2_) are the isolated energies of bare Zr_3_C_2_ and the functional groups, respectively. Specifically, relative to the O_2_, F_2_, and Cl_2_ molecules, $E\left(S_{2}\right)$ represents twice the energy of a single S atom. The results of the formation energies are also shown with the corresponding structure in Figure 2. It is shown in Figure 2 that all the formation energies are negative, confirming their energetic stability. Additionally, Figure 2 reveals that surface functional groups in Zr_3_C_2_T_2_ (T = O, S, F, Cl) preferentially occupy CCP sites, which exhibit the lowest formation energies among the three considered sites. This observation aligns with findings previously reported in the literature [63].

For the bare Zr_3_C_2_ and Zr_3_C_2_T_2_ with the most stable configurations (CPP sites), the optimized lattice parameters and formation energies were summarized for comparison in Table 1. It is revealed in Table 1 that the formation energies of surface-functionalized Zr_3_C_2_T_2_ (−6.173 eV for Zr_3_C_2_O_2_ and −5.605 eV for Zr_3_C_2_F_2_) located in the second period of the periodic table are lower than those located in the third period (−4.169 eV for Zr_3_C_2_S_2_ and −4.010 eV for Zr_3_C_2_Cl_2_).

In addition, the work functions of Zr_3_C_2_ and Zr_3_C_2_T_2_ were calculated, which are also listed in Table 1. The results show that the work function is directly proportional to the formation energy of the functional groups: the greater the absolute value of formation energy, the larger the work function. The obtained work functions are in the following order: Zr_3_C_2_ < Zr_3_C_2_Cl_2_ < Zr_3_C_2_F_2_ < Zr_3_C_2_S_2_ < Zr_3_C_2_O_2_.

### 3.2. Electronic Properties

**Figure 3:**
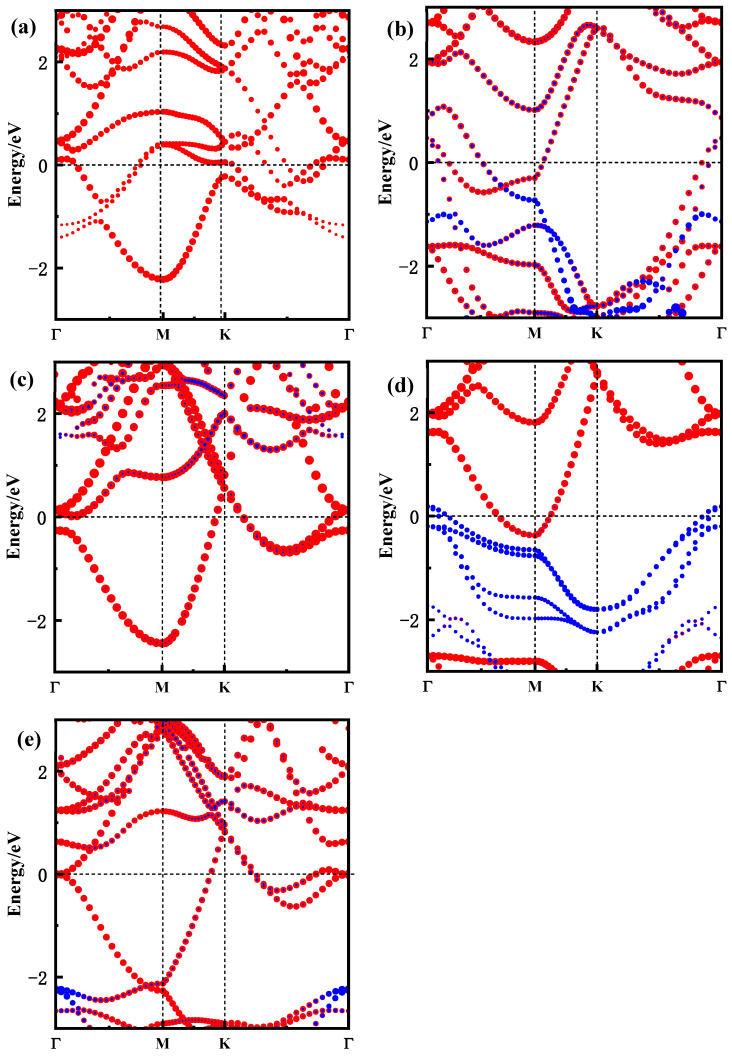
Band structure of Zr_3_C_2_ and Zr_3_C_2_T_2_ along the high-symmetry path Γ(0 0 0)-M(0.5 0 0)-K(0.333 0.333 0)-Γ(0 0 0). (**a**) Zr_3_C_2_; (**b**) Zr_3_C_2_O_2_; (**c**) Zr_3_C_2_F_2_; (**d**) Zr_3_C_2_S_2_; and (**e**) Zr_3_C_2_Cl_2_. (The red and blue lines represent the energy band contributed by Zr_3_C_2_ and functional group T_2_, respectively).

The band structure is a crucial factor in understanding the electronic properties of materials. For the Zr_3_C_2_T_2_ MXene, the electronic properties are closely linked to the surface functional groups. To analyze the impact of these functional groups on the electronic behavior of the MXene, the high symmetry path Γ(0 0 0)-M(0.5 0 0)-K(0.333 0.333 0)-Γ(0 0 0) was selected to calculate the band structure of Zr_3_C_2_T_2_ (T = O, S, F, Cl), and the results are shown in Figure 3. It is indicated that the functional groups primarily affect the valence band of Zr_3_C_2_T_2_, while their impact on the conduction band is minimal. Compared to the fluorine and chlorine functional groups, the band structure near the Fermi level is more affected by the O and S functional groups. When Zr_3_C_2_ is functionalized by O and S, as shown in Figure 3a,c, the band structures near the Fermi level are the result of functional group atoms, and even new band structures are generated near the Fermi level. However, in the band structure of Zr_3_C_2_F_2_ and Zr_3_C_2_Cl_2_, it is clear in Figure 3b,d that the contribution of functional groups near the Fermi level is almost invisible, and the contribution of functional groups is mainly in the valence band where the energy is much lower than the Fermi level. In addition, an interesting phenomenon was found in which the band structure of Zr_3_C_2_O_2_ and Zr_3_C_2_S_2_ had a clear tendency to move towards the conduction band, in contrast to Zr_3_C_2_F_2_ and Zr_3_C_2_Cl_2_. This is mainly due to the stronger hybridization of the O and S functional groups with Zr_3_C_2_, which enhances the expansibility of atomic orbitals of the valence band near the Fermi level.

**Figure 4:**
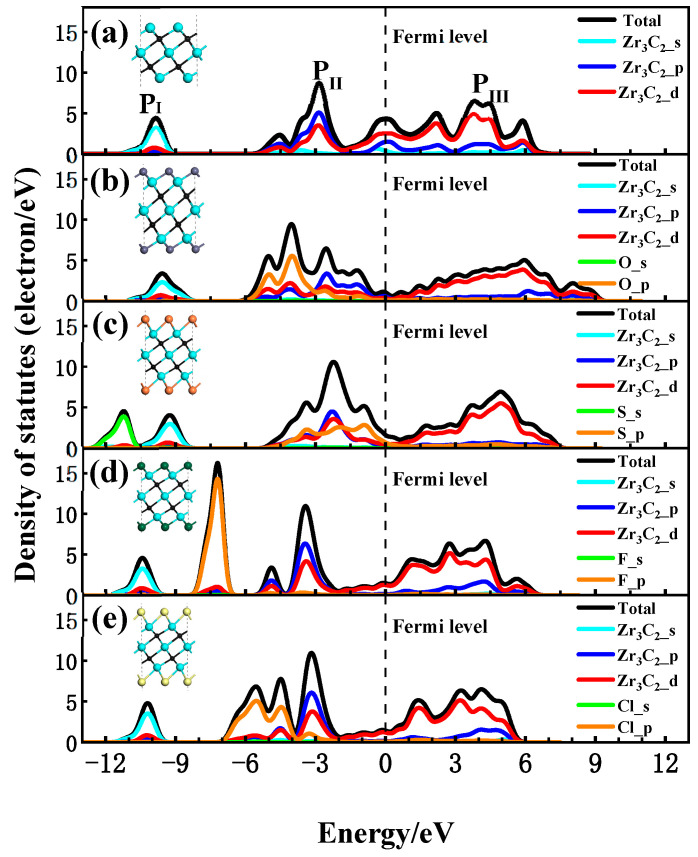
Density of states of Zr_3_C_2_ and Zr_3_C_2_T_2_ with corresponding structural diagram: (**a**) bare Zr_3_C_2_; (**b**) Zr_3_C_2_O_2_; (**c**) Zr_3_C_2_S_2_; (**d**) Zr_3_C_2_F_2_; and (**e**) Zr_3_C_2_Cl_2_.

To discuss the interaction between the functional groups and Zr_3_C_2_, the total (DOS) and partial density of states (PDOS) for the bare Zr_3_C_2_ and functionalized Zr_3_C_2_T_2_ with the corresponding structural diagram are shown in Figure 4. In the DOS and PDOS of bare Zr_3_C_2_ in Figure 4a, a broader hybridization peak (0–6 eV) appears in the conduction band above the Fermi level due to the hybridization of Zr and C, indicating a strong Zr-C bond. There are two main peaks in the valence band, one contributed by the Zr_3_C_2_-p orbitals (−10 eV) and the other by the combined Zr_3_C_2_-p and Zr_3_C_2_-d orbitals (−3 eV). In addition, a large peak at the Fermi level may induce instabilities in bare Zr_3_C_2_, suggesting that Zr_3_C_2_ tends to be functionalized by terminal groups [61]. As can be seen in Figure 4b–e, different functional groups have different effects on the DOS of Zr_3_C_2_T_2_. The results show that when Zr_3_C_2_ was functionalized by O, F, S, and Cl, the DOS near the Fermi level was affected by the p orbital of the functional groups, while the s orbital of the functional groups only affected the valence band away from the Fermi level. In the PDOS of Zr_3_C_2_O_2_ and Zr_3_C_2_S_2_, as shown in Figure 4b,c, it is the hybridization between the p orbitals of the functional groups (O-p and S-p) and the Zr_3_C_2_-d orbitals that enhances the extensibility of the hybridization peak originally located at −3 eV and made the DOS near the Fermi level move towards the conduction band. This phenomenon observed in Figure 4b,c aligns with the band structures of Zr_3_C_2_O_2_ and Zr_3_C_2_S_2_, which exhibit a distinct tendency to shift toward the conduction band. In addition, the broader hybridization peaks in the conduction band are also affected by the oxygen and sulfur functional groups. Because of the effect of the functional groups, the contribution of the Zr_3_C_2_-p orbitals to the wide peaks is reduced and shows stronger localization at −2 eV~0 eV. However, it can be seen in Figure 4d,e that when the Zr_3_C_2_ MXene was functionalized by F and Cl, the hybrid peak between the functional groups and Zr_3_C_2_ was not found.

**Figure 5:**
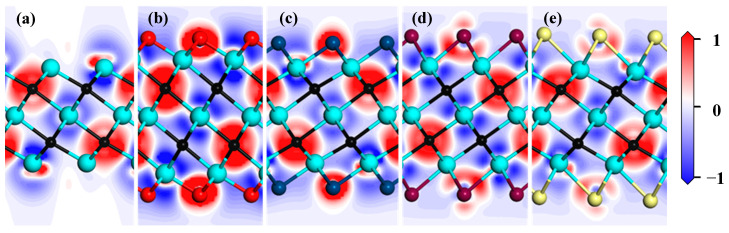
Differential density charge of bare Zr_3_C_2_ and Zr_3_C_2_T_2_ (T = O, S, F, Cl): (**a**) bare Zr_3_C_2_, (**b**) Zr_3_C_2_O_2_, (**c**) Zr_3_C_2_F_2_, (**d**) Zr_3_C_2_S_2_, and (**e**) Zr_3_C_2_Cl_2_ (red represents electron depletion and blue represents accumulation).

Differential charge density is an important way to analyze interatomic bonding and charge transfer. To discuss the effect of functional groups on charge transfer in the Zr_3_C_2_ MXene, differential charge density for the bare Zr_3_C_2_ and functionalized Zr_3_C_2_T_2_ are shown in Figure 5, in which the electron depletion and accumulation are denoted in red and blue, respectively. The result in Figure 5 shows that the charges accumulated around the C atoms, and the charges depleted around the surface Zr atoms in the bare Zr_3_C_2_. This suggests that bare Zr_3_C_2_ exhibits ionic bonding characteristics, with strong directional bonds forming between the Zr and C atoms. It is noteworthy that a small amount of charge accumulates on the surface of the low coordination Zr atoms, while the charge is consumed on the side of these Zr atoms near the C atom. When Zr_3_C_2_ is functionalized, the functional group atoms participate in charge transfer. Compared with the Zr_3_C_2_O_2_ and Zr_3_C_2_S_2_, the charge transfers between the functional group atoms of Zr_3_C_2_F_2_ and Zr_3_C_2_Cl_2_ with the surface Zr atoms are weaker.

Moreover, the Mulliken population analysis offers a quantitative measure for evaluating bonding characteristics. As shown in Table 2, the analysis reveals a charge transfer of 1.56 electrons from the Zr to C atoms in the bare Zr_3_C_2_, of which 0.9 electrons are contributed by the central Zr atom. Due to the influence of the functional groups, the charge transfers between the Zr and C atoms increased (1.60–1.66 electrons), indicating that the Zr-C bond is stronger. In addition, when Zr_3_C_2_ is functionalized by oxygen and sulfur, there is a strong charge transfer between the functional group atoms and the surface Zr atoms. However, when Zr_3_C_2_ is functionalized by fluorine and chlorine, the charge transfer between the functional group atoms and the surface Zr atoms is weak. These discrepancies may be caused by atomic electronegativity.

### 3.3. Lithium Storage Performance

**Figure 6:**
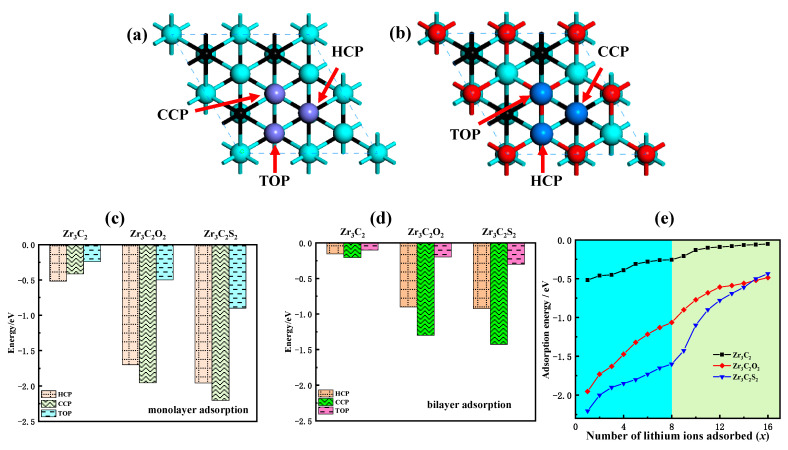
Lithium storage properties of bare Zr_3_C_2_ and Zr_3_C_2_T_2_. (**a**,**b**) The potential adsorption sites of lithium ions on bare Zr_3_C_2_ and Zr_3_C_2_T_2_ surfaces, respectively; (**c**,**d**) The monolayer and bilayer adsorption energies of lithium ions at HCP, CCP, and TOP sites, respectively; (**e**) The relationship between the number of adsorbed lithium ions (x) and the adsorption energy.

Monolayer adsorption and multilayer adsorption are studied in the study of the lithium storage performance of Zr3C2T2. The potential sites of monolayer and multilayer adsorption of lithium ions on the Zr3C2 surface are shown in Figure 6a,b.

To identify the most stable adsorption site for lithium ions on the surface of both bare Zr_3_C_2_ and functionalized Zr_3_C_2_T_2_, the adsorption energy at each potential site was determined using Equation (2) [47]:

(2) $${\Delta E}_{ab}=E_{sub+xLi}-E_{sub}-xE\left(Li\right)$$

where *E*_sub+xLi_ denotes the total energy of bare Zr_3_C_2_ or Zr_3_C_2_T_2_ with lithium ions adsorbed, x represents the number of adsorbed lithium ions, *E*_sub_ is the energy of isolated bare Zr_3_C_2_ or Zr_3_C_2_T_2_, and *E*(Li) corresponds to the average energy of lithium ions. Figure 6c–d present the monolayer and bilayer adsorption energies of lithium ions at various adsorption sites. For the bare Zr_3_C_2_, the monolayer adsorption energies (Figure 6d) are −0.52 eV (HCP), −0.41 eV (CCP), and −0.24 eV (TOP). In comparison, the bilayer adsorption energies (Figure 6e) are −0.16 eV (HCP), −0.21 eV (CCP), and −0.10 eV (TOP). Compared with bare Zr_3_C_2_, the adsorption energy of lithium ions at the Zr_3_C_2_ functionalized by O and S functional groups is reduced. By contrast, the adsorption energy of Zr_3_C_2_F_2_ and Zr_3_C_2_Cl_2_ on lithium ions increased to a positive value, indicating that the Zr_3_C_2_T_2_ functionalized by fluoride and chlorine could not stably adsorb lithium ions. In addition, the most stable adsorption site of lithium-ion is also affected by the terminals. The most stable adsorption site for lithium ions in the monolayer adsorption of bare Zr_3_C_2_ is the HCP site, and the most stable adsorption site in the bilayer adsorption of bare Zr_3_C_2_ is the CCP site. When Zr_3_C_2_ is functionalized with O and S, the monolayer adsorption of lithium ions starts at the CCP site. For bilayer adsorption in Zr_3_C_2_O_2_, the HCP site is identified as the most stable adsorption site, which is like the switching of adsorption sites in the bare Zr_3_C_2_ monolayer adsorption and bilayer adsorption. However, the most stable adsorption site in the bilayer adsorption of Zr_3_C_2_S_2_ is still the CCP site, which is not like the switching of the adsorption site with bare Zr_3_C_2_.

To further understand the influence of the functional groups on the lithium storage properties of Zr_3_C_2_T_2_, the relationship between the amount of adsorbed lithium ions (x) and the adsorption energy for both monolayer and bilayer adsorption was investigated. The findings, presented in Figure 6e, show that the curve within the sky blue area represents the variation in adsorption energy during monolayer adsorption (x ≤ 8), while the curve in the light green region corresponds to the bilayer adsorption (9 ≤ x ≤ 16). The results indicate that the adsorption energy increases as the number of adsorbed lithium ions rises. The adsorption energy increases from −0.52 eV to −0.05 eV for bare Zr_3_C_2_, from −1.95 eV to −0.49 eV for Zr_3_C_2_O_2_, and from −2.20 eV to −0.44 eV for Zr_3_C_2_S_2_. In addition, the adsorption energy curve of each layer always rises steeply and then gently. Compared with the adsorption energy curve of monolayer adsorption, the front part of the adsorption curve of bilayer adsorption is steeper. This phenomenon is easily inferred that bare Zr_3_C_2_ makes it difficult to continue the three-layer adsorption.

**Figure 7:**
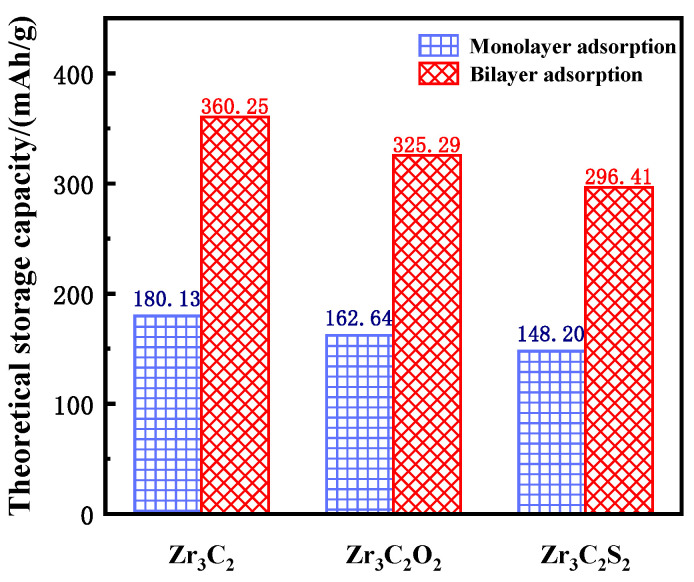
The theoretical storage capacity of bare Zr_3_C_2_ and Zr_3_C_2_T_2_.

The theoretical lithium storage capacity of bare and functionalized Zr_3_C_2_T_2_ (T = O, S) was calculated using Equation (3) [64]:

(3) $$C=\frac{x\times z\times F\times 10^{3}}{4W}$$

where *z* represents the valence number (*z* = 1 for Li), *F* is the Faraday constant (26.810 Ah/mol), and *W* is the atomic mass (297.68 g/mol for Zr_3_C_2_, 329.68 g/mol for Zr_3_C_2_O_2_, and 361.80 g/mol for Zr_3_C_2_S_2_). In Equation (3), 4*W* is used because a 2 × 2 × 1 supercell was constructed to investigate the lithium storage properties. The theoretical lithium storage capacity of single-layer adsorption and double-layer adsorption was calculated separately, and the results are shown in Figure 7. The sky blue squares represent the theoretical capacity of monolayer adsorption, and the red oblique squares represent the theoretical capacity of bilayer adsorption. The theoretical lithium storage capacities of bare Zr_3_C_2_, Zr_3_C_2_O_2_, and Zr_3_C_2_S_2_ are 180.13 mAh/g, 162.64 mAh/g, and 148.20 mAh/g for monolayer adsorption and 360.25 mAh/g, 325.29 mAh/g, and 296.41 mAh/g for bilayer adsorption, respectively. Although the theoretical capacity of the bare Zr_3_C_2_ is higher than that of the functionalized Zr_3_C_2_T_2_ (Zr_3_C_2_O_2_ and Zr_3_C_2_S_2_), the application of the functionalized Zr_3_C_2_T_2_ in lithium storage is more promising because it is difficult for the bare Zr_3_C_2_ to continue the three-layer adsorption, while Zr_3_C_2_O_2_ and Zr_3_C_2_S_2_ can continue adsorption.

## 4. Conclusions

Using DFT calculations, the effects of surface terminals on the structure, electronic properties, and lithium storage properties of Zr_3_C_2_T_2_ were studied. Functional groups from the oxygen group (T = O, S) and halogen family (T = F, Cl) were considered in this paper. The CCP site was identified as the most stable adsorption site on the Zr_3_C_2_T_2_ surface. The structural properties of Zr_3_C_2_T_2_ were only slightly affected by the surface functional groups, which mainly showed that the presence of functional groups increased the bond length of Zr1-C. The electronic properties of Zr_3_C_2_T_2_ were greatly affected by the O and S functional groups. It was shown that the O and S functional groups had strong hybridization with Zr_3_C_2_ in the valence band near the Fermi level and generated a new band, which made the DOS move towards the conduction band. However, the halogen functional groups (F, Cl) had little effect on the electronic properties of Zr_3_C_2_. The results of the differential charge density and Mulliken population indicated that the charge transfer between oxygen group atoms and Zr_3_C_2_ was more than the counterpart between halogen atoms and Zr_3_C_2_. The adsorption energy of lithium ions on the Zr_3_C_2_T_2_ functionalized by oxygen and sulfur functional groups was reduced. By contrast, the Zr_3_C_2_T_2_ functionalized by F and Cl could not stably adsorb lithium ions. Additionally, surface functionalization altered the initial adsorption site of lithium atoms. In monolayer adsorption, the most favorable site for lithium ion adsorption was the HCP site for bare Zr_3_C_2_, while it shifted to the CCP site for both Zr_3_C_2_O_2_ and Zr_3_C_2_S_2_. For the bilayer adsorption, the most suitable adsorption sites were CCP for bare Zr_3_C_2_, HCP for Zr_3_C_2_O_2_, and CCP for Zr_3_C_2_S_2_. It was revealed that the adsorption energy of both the monolayer and bilayer always rose steeply and then gently. It also showed that bare Zr_3_C_2_ made it difficult to continue the three-layer adsorption. The theoretical capacities of the bare Zr_3_C_2_ (360.25 mAh/g), Zr_3_C_2_O_2_ (325.29 mAh/g), and Zr_3_C_2_S_2_ (296.41 mAh/g) were predicted.

In future work, the effects of other functional groups and mixed functional groups on the lithium storage performance of the Zr_3_C_2_ MXene, the migration of lithium on the MXene surface, and its diffusion energy barrier will be theoretically investigated. In addition, experiments on the effects of Zr_3_C_2_ and functional groups on lithium storage will be conducted to validate the theoretical calculations.

## Figures and Tables

**Table 1:** The optimized structural parameters, formation energy, and work function at CPP sites of Zr_3_C_2_ and Zr_3_C_2_T_2_.

Zr_3_C_2_T_2_	a (Å)	h (Å)	Formation Energy (eV)	Work Function (eV)
Zr_3_C_2_	3.330	5.100	/	3.83
Zr_3_C_2_O_2_	3.322	7.333	−6.173	8.16
Zr_3_C_2_S_2_	3.320	8.774	−4.169	6.61
Zr_3_C_2_F_2_	3.328	8.394	−5.605	5.44
Zr_3_C_2_Cl_2_	3.325	8.845	−4.010	4.65

**Table 2:** Mulliken population of bare Zr_3_C_2_ and Zr_3_C_2_T_2_.

Zr_3_C_2_T_2_	Species	s	p	d	Total	Charge (e)
Zr_3_C_2_	C	1.49	3.29	0	4.78	−0.78
	C	1.49	3.29	0	4.78	−0.78
	Zr	2.51	6.49	2.67	11.67	0.33
	Zr	2.51	6.49	2.67	11.67	0.33
	Zr	2.14	6.31	2.64	11.1	0.9
Zr_3_C_2_O_2_	C	1.49	3.31	0	4.8	−0.8
	C	1.49	3.31	0	4.8	−0.8
	Zr	2.18	6.32	2.41	10.91	1.09
	Zr	2.18	6.32	2.41	10.91	1.09
	Zr	2.1	6.4	2.7	11.2	0.8
	O	1.87	4.81	0	6.69	−0.69
	O	1.87	4.81	0	6.69	−0.69
Zr_3_C_2_S_2_	C	1.52	3.28	0	4.8	−0.8
	C	1.52	3.28	0	4.8	−0.8
	Zr	2.33	6.45	2.61	11.38	0.62
	Zr	2.33	6.45	2.61	11.38	0.62
	Zr	2.19	6.46	2.57	11.22	0.78
	S	1.87	4.35	0	6.21	−0.21
	S	1.87	4.35	0	6.21	−0.21
Zr_3_C_2_F_2_	C	1.48	3.35	0	4.83	−0.83
	C	1.48	3.35	0	4.83	−0.83
	Zr	2.23	6.31	2.5	11.03	0.97
	Zr	2.23	6.31	2.5	11.03	0.97
	Zr	2.14	6.42	2.66	11.23	0.77
	F	1.95	5.57	0	7.52	−0.52
	F	1.95	5.57	0	7.52	−0.52
Zr_3_C_2_Cl_2_	C	1.49	3.32	0	4.81	−0.81
	C	1.49	3.32	0	4.81	−0.81
	Zr	2.31	6.39	2.62	11.32	0.68
	Zr	2.31	6.39	2.62	11.32	0.68
	Zr	2.17	6.42	2.64	11.23	0.77
	Cl	1.94	5.31	0	7.25	−0.25
	Cl	1.94	5.31	0	7.25	−0.25

## Data Availability

The original contributions presented in the study are included in the article; further inquiries can be directed to the corresponding author.
